# Can performance-based incentives improve motivation of nurses and midwives in primary facilities in northern Ghana? A quasi-experimental study

**DOI:** 10.3402/gha.v9.32404

**Published:** 2016-10-13

**Authors:** Gifty Apiung Aninanya, Natasha Howard, John E. Williams, Benjamin Apam, Helen Prytherch, Svetla Loukanova, Eunice Karanja Kamara, Easmon Otupiri

**Affiliations:** 1 School of Public Health, Kwame Nkrumah University of Science and Technology, Kumasi, Ghana; 2 Department of Global Health and Development, London School of Hygiene and Tropical Medicine, London, UK; 3 Navrongo Health Research Centre, Navrongo, Ghana; 4 Department of Statistics, Bolgatanga Polytechnic, Bolgatanga, Ghana; 5 Swiss Tropical and Public Health Institute, University of Basel, Basel, Switzerland; 6 Department of General Practice and Health Services Research, Heidelberg University Hospital, Heidelberg, Germany; 7 Department of Philosophy, Religion and Theology, Moi University, The Eldoret, Kenya

**Keywords:** Ghana, performance-based incentives, motivation, constructs of motivation, health worker

## Abstract

**Background:**

Lack of an adequate and well-performing health workforce has emerged as the biggest barrier to scaling up health services provision in sub-Saharan Africa. As the global community commits to the Sustainable Development Goals and universal health coverage, health workforce challenges are critical. In northern Ghana, performance-based incentives (PBIs) were introduced to improve health worker motivation and service quality.

**Objective:**

The goal of this study was to determine the impact of PBIs on maternal health worker motivation in two districts in northern Ghana.

**Design:**

A quasi-experimental study design with pre- and post-intervention measurement was used. PBIs were implemented for 2 years in six health facilities in Kassena-Nankana District with six health facilities in Builsa District serving as comparison sites. Fifty pre- and post-intervention structured interviews and 66 post-intervention in-depth interviews were conducted with health workers. Motivation was assessed using constructs for job satisfaction, pride, intrinsic motivation, timelines/attendance, and organisational commitment. Quantitative data were analysed to determine changes in motivation between intervention and comparison facilities pre- and post-intervention using STATA™ version 13. Qualitative data were analysed thematically using NVivo 10 to explore possible reasons for quantitative findings.

**Results:**

PBIs were associated with slightly improved maternal health worker motivation. Mean values for overall motivation between intervention and comparison health workers were 0.6 versus 0.7 at baseline and 0.8 versus 0.7 at end line, respectively. Differences at baseline and end line were 0.1 (*p*=0.40 and *p*=0.50 respectively), with an overall 0.01 difference in difference (*p*=0.90). Qualitative interviews indicated that PBIs encouraged health workers to work harder and be more punctual, increasing reported pride and job satisfaction.

**Conclusions:**

The results contribute evidence on the effects of PBIs on motivational constructs among maternal health workers in primary care facilities in northern Ghana. PBIs appeared to improve motivation, but not dramatically, and the long-term and unintended effects of their introduction require additional study.

## Introduction

Globally, the World Health Organization (WHO) and others have advocated extensively for motivated health workers to provide high-quality maternal health care and attain national and global health goals (1). High-quality maternal care cannot be delivered effectively in sub-Saharan Africa unless demotivation of nurses and midwives is comprehensively tackled (2). *Motivation*, the ‘willingness to exert and maintain an effort towards organisational goals’ (3), can be intrinsic or extrinsic. Intrinsically motivated health workers conduct activities because they lead to personal satisfaction, whereas extrinsic motivation works because of external rewards associated with achieving objectives (4). Intrinsic motivators include feelings of empathy for patients or pride in doing one's best. Extrinsic motivators include verbal recognition from employers and peers, gifts, and financial rewards for achieving recognised targets. Theories of motivation (e.g. Adam's equity theory, Vroom's expectancy theory) are intrinsic in nature, whereas needs-based theories (e.g. Maslow's hierarchy of needs, McClelland's acquired needs theory) are extrinsic (5).

A combination of intrinsic and extrinsic motivators has been shown to improve health worker motivation (6), retention, and performance (2). Improved performance can provide a sense of achievement, resulting in greater motivation, but it is difficult to achieve when little motivation initially exists (7). Thus, motivation and performance can be mutually reinforcing (3, 8). Low health worker motivation is characterised by poor practices, including negative attitudes towards clients (9), lateness and absenteeism (10–12), high turnover (13), and health worker migration (14). Both financial (15) and non-financial (16–18) performance-based incentives (PBIs), used interchangeably with *performance-based pay*, *performance pay*, *pay-for-performance* (P4P), and *results-based financing*, can strengthen health worker motivation and performance in low-income countries (18–20). Several studies indicate positive effects of PBIs, particularly among health workers with lower levels of intrinsic motivation (21). An US study of 348 employees from nine organisations showed that PBIs enhanced motivation (22). Kuwait workers demonstrated increased motivation with extrinsic rewards (23). In sub-Saharan Africa, evaluations linking provider payment to defined outcomes showed improved service coverage and quality-of-care target achievements (20, 24, 25). A year-long PBI pre-pilot in three Burkina Faso health districts demonstrated improvements in quantity and quality scores for maternal health services (26). In South Africa and the Democratic Republic of Congo, financial and non-financial incentives increased health worker motivation (27, 28). Use of financial incentives to motivate healthcare workers has had varying results, indicating that financial incentives without complementary non-financial incentives rarely improve health worker motivation and performance in the long term (10, 29). Despite successes, in some countries health workers were frustrated when incentives were relatively low compared with workload increases or if distribution was perceived as non-transparent or inequitable (30, 31). Multifaceted motivational interventions are therefore recommended, influencing different motivators at the same time, which can be evaluated through motivational constructs, for example, job satisfaction and self-efficacy (32, 33).

Although maternal health worker competence and motivation appear essential to achieving Sustainable Development Goal 3 (34), poor health worker motivation exists in Ghana (12, 29, 32, 35, 36) and other African health systems (37, 38). This is partly due to low remuneration. Ghana has just over 20,000 professional nurses. Each year about 400 enter the job market, but many also leave (e.g. 300 in 2015), and most work in urban areas (39). For example, the Greater Accra Region comprises 19% of Ghana's population with about 31% of registered nurses, whereas the three northern regions comprise 18% of Ghana's population with 16% of registered nurses (40). Living costs are high, yet public-sector nurses only earn approximately US$400 per month after tax and frequently face delayed salary payments. Vehicle hire purchase and deprived-area incentive schemes have been introduced to try to increase health worker motivation and performance but remain unequally and inefficiently applied (32, 41, 42). Health workers may be stuck in rural postings for many years. Maintaining health worker motivation without freedom of geographical location is challenging (36).

Although evidence indicates that PBIs have potential to improve health worker motivation, previous research was conducted in different public and private settings and study populations (e.g. physicians and office workers), and several lacked baseline measurements, credible comparison groups, or before and after comparisons. Studies have generally focused on associations of PBIs and motivation, without examining their effects on specific motivation constructs, for example, job satisfaction and intrinsic motivation. While constructs have been defined that collectively measure health worker motivation (43–45), no evidence yet exists of the effects of PBIs on motivation constructs among nurses and midwives in low- and middle-income countries.

Given the limited evidence associating PBIs with improved health worker motivation constructs, this study aimed to determine the impact of PBIs on the motivation of nurses and midwives in primary-level health facilities in two districts of northern Ghana. Baseline studies showed that poor health worker motivation was affecting provision of maternal health services (36, 46). The objectives were to use quantitative and qualitative methods to explore the effects of PBIs on 1) job satisfaction, 2) intrinsic motivation, 3) pride, 4) organisational commitment, and 5) timeliness and attendance.

## Methods

### Setting and subjects

The study was conducted in Kassena-Nankana (KND) and Builsa districts in the Upper East Region of northern Ghana (47, 48). KND has eight health centres, two private clinics, 27 Community-based Health Planning and Services (CHPS) compounds, whereas Builsa District has six health centres and 15 CHPS compounds (49). The health centre is the first point of contact between service users and the health system. In this region, a health centre typically serves a population of approximately 20,000 and is managed by a clinical officer or medical assistant (50). Health workers provide basic preventive and curative services, minor surgical procedures, and maternal and child health care. Health centre nurses and midwives are primarily responsible for maternal and neonatal health, providing skilled antenatal, childbirth, postnatal and family planning care, referrals, and health education. Each district has a referral hospital providing comprehensive emergency obstetric care (51). Approximately 207 and 100 health workers serve health facilities in KND and Builsa District, respectively. Health facilities are challenged by inadequate health personnel, heavy workload, poor motivation (36, 52), a doctor/patient ratio of 1:75,488, and a nurse/patient ratio of 1:5,245.

### Study design

A mixed-method design was selected for the 2-year study, with baseline and end line measurement through quasi-experimental quantitative cohort survey and end line semi-structured interviews. This approach was employed to allow for triangulation of results using different methods and exploration of changes over time (53, 54). This study was conducted under the European Union-funded QUALMAT project to improve maternal and neonatal healthcare quality in Ghana, Tanzania, and Burkina Faso (55, 56). Motivation constructs of job satisfaction, intrinsic motivation, pride, timeliness, and attendance were defined based on a literature review for constructs used successfully in African settings (44) and measurement tools developed during piloting for the three countries (44, 45).

All primary health centres in KND and Builsa District that offered antenatal and delivery care were included, with six KND facilities allocated as intervention and six Builsa District facilities as comparison. All facilities were comparable in terms of infrastructure, equipment, staff, and obstetric services provided. All nurses and midwives providing maternal and neonatal care in participating health centres, who provided informed consent, participated in the cohort study (*n*=50). As it was not possible to include additional facilities in other districts to increase the sample size, and numbers were small, no sampling was conducted. Qualitative study participants were recruited purposefully (*n*=50), using homogeneous sampling to include those most able to provide insights into phenomena of interest (53).

### Intervention

PBIs for nurses and midwives were introduced from July 2012 to March 2014 (44, 46). Aiming to improve maternal health worker motivation and performance in six KND sites, these PBIs consisted of financial and non-financial awards provided to the best-performing health workers at biannual ceremonies. Health workers achieving scores of 70–100%, as determined by a committee, qualified for an award. Awards included small monthly allowances of approximately US$20, refrigerators, televisions, microwaves, blenders, saucepans, tea kettles, cloths, or certificates of recognition. Award ceremonies were attended by the District Director of Health Services for KND West, Municipal Director of Health Services for KND East Municipality, public health nurses, medical assistants, the director of the Navrongo Health Research Centre (NHRC), and senior NHRC staff.

A PBI review committee, comprising an official from the Upper East Regional Health Directorate, district directors, district public health nurses, and three QUALMAT staff, developed performance assessment criteria and indicators and standardised awards through consultation with health workers, regional and district health directorates, and selected awardees. A formal review process identified best performers in each facility by summing scores for proportions of service users who attended at least four antenatal visits, received tetanus vaccinations, had haemoglobin and blood pressure levels continuously checked, established their HIV status, received appropriate antenatal and labour referrals, and delivered at a facility with skilled attendance. All issues associated with incentives were agreed on with health workers and managers. Additionally, supervisors were interviewed and health worker output records monitored to ensure that selected health workers were ‘hardworking’ as per agreed definitions.

### Data collection

#### Tools and researchers

The structured questionnaire comprised 42 questions, including 1) socio-demographic characteristics and 2) motivation constructs. Socio-demographic characteristics were sex, age, job title, years in maternal health, and years in facility. Motivation constructs were pride, job satisfaction, intrinsic motivation, timeliness, and attendance, as drawn from Zambian and Kenyan research and used in other QUALMAT studies (33, 45). Reported effects were measured using a four-point Likert scale (i.e. strongly agree, agree, disagree, and strongly disagree) developed from the literature (44). Face validity was assessed by five external motivation experts, who reviewed the effectiveness of each question at measuring motivation. The interview guide was developed from relevant literature and study objectives and validated through stakeholder and expert review. Interviews included 14 questions on demographic information, motivation constructs, and experiences with PBIs. Both qualitative and quantitative tools were piloted among 70 health workers in Bongo District, outside the study area (i.e. 50 for quantitative tools and 20 for qualitative tools), who were additionally asked to consider whether tools would measure what was intended. Tools were then revised as needed. Two experienced research assistants were trained for 5 days on study objectives, interview techniques, questionnaire content, probing, and rapport building.

#### Quantitative surveys

Researchers collected informed consent and conducted pre- and post-intervention surveys with all nurses and midwives in 12 primary health centres in KND and Builsa District in April–May 2011 and May–August 2014. Surveys were conducted face to face at health centres and lasted about 15 min. The first author supervised research assistants and checked completed questionnaires at the end of each day to ensure accuracy and completeness and address any issues.

#### Qualitative interviews

After collecting informed consent, the first author and two research assistants conducted semi-structured interviews in both districts with health facility managers, district-level staff, nurses, and midwives. Interviews focused on participant perspectives, feelings, and experiences (57); were conducted in English at locations selected by participants; took approximately 40 min; and were audio-recorded and transcribed by the first author and research assistants.

### Data analysis

#### Quantitative

Survey data were entered using Epidata and analysed in STATA 13. Descriptive statistics summarised demographic variables (e.g. age, years in current facility). Factor analysis was used to obtain a score summarising all variables relating to each motivation construct, to evaluate changes in intervention and comparison sites over time. Answers were summed to generate individual propensity scores for each construct (i.e. between 0 for lowest motivation and 1 for highest). Thus, scores above 0.7 indicated high satisfaction, 0.5–0.6 moderate satisfaction, and below 0.5 low satisfaction. With 14 items scaled, the reliability coefficient was 0.7041. A difference in difference (DiD) analysis was conducted to estimate PBI effects; comparing pre- and post-intervention differences in motivation construct scores between intervention and comparison arms. *P*-values were considered significant at 0.05 or less.

#### Qualitative

Transcripts were exported into NVivo 10 and analysed thematically (58). First, transcripts were read critically. Second, deductive coding used motivation constructs (i.e. job satisfaction, health worker pride, organisational commitment, intrinsic motivation, timeliness, and attendance). Third, inductive coding identified additional subthemes (59–62). Inclusion of narratives from different levels of the health system (i.e. midwives, nurses, medical assistants, district public health nurses, and directors) in the two study arms increased validity.

### Ethics

The study was approved by the institutional review boards of the Kwame Nkrumah University of Science and Technology and NHRC in Ghana (reference NHRCIRB 085) and the ethics committee of the University of Heidelberg in Germany (reference S173/2008). Written and oral informed consent were obtained from all participants prior to inclusion. Reporting followed CONSORT guidelines.

## Results

Table 1 shows the baseline characteristics for 50 cohort participants, 25 in the six intervention sites and 25 in the six comparison sites. Fifty-two percent of intervention and sixty-four percent of comparison participants were aged 40–49 years, whereas 75% of intervention and 65% of comparison participants were midwives. Forty-six percent of intervention and fifty-eight percent of comparison participants had worked in maternal health for 11 or more years and 58% of intervention and 73% of comparison participants had worked at their present health facility for 1–5 years.

**Table 1:** Baseline demographic characteristics of cohort survey participants

Variable	Intervention (%)	Comparison (%)
Sex		
Women	25 (100)	25 (100)
Men	–	–
Age group		
20–39	11 (44)	9 (36)
40–59	13 (52)	16 (64)
60 +	1 (4)	–
Profession		
Community health nurse	5 (21)	6 (23)
Midwife	18 (75)	17 (65)
Other (i.e. medical assistant, public health nurse)	1 (4)	3 (12)
Years in maternal health		
<1	4 (17)	1 (4)
1–5	4 (17)	5 (19)
6–10	5 (21)	5 (19)
11+	11 (46)	15 (58)
Years in facility		
<1	6 (25)	3 (12)
1–5	14 (58)	19 (73)
6–10	4 (17)	3 (12)
11+	–	1 (4)

Table 2 shows that 66 staff from intervention sites (32) and comparison sites (32) participated in qualitative interviews, 92% of whom were women, 73% nurses or midwives, 18% facility managers, and 9% district-level staff. During 2012–2014, health workers in the six intervention health centres received a total of US$480 in financial incentives, while 25 health workers additionally received non-financial incentives.

**Table 2:** Demographic characteristics of qualitative study participants

Demographics	Intervention (%)	Comparison (%)	Total (%)
Position			
Health worker (midwife) from cohort sample	22 (67)	22 (67)	44 (67)
Health worker (nurse) from cohort sample	2 (6)	2 (6)	4 (6)
Facility manager	6 (18)	6 (18)	12 (18)
District-level staff	3 (9)	3 (9)	6 (9)
Gender			
Women	30 (91)	31 (94)	61 (92)
Men	3 (9)	2 (6)	5 (8)

Health worker comments are presented under deductive themes of overall motivation, job satisfaction, pride, intrinsic motivation, timeliness and attendance, and organisational commitment. Inductive subthemes are included where appropriate.

### Overall motivation

Table 3 shows quantitative results. At baseline, combined overall average motivation scores among health workers in intervention and comparison facilities were 0.63 and 0.73, respectively, a difference of 0.11 (*p*=0.40). At end line, overall motivation increased to 0.80 among intervention health workers and remained at 0.72 for comparison health workers, which was not significantly different (*p*=0.50). The DiD value between intervention and comparison groups at baseline and end line was 0.01 (*p*=0.90).

**Table 3:** Motivational construct values in intervention and comparison facilities at baseline and end line

	Baseline						End line						DiD	
			
	Intervention		Comparison				Intervention		Comparison					
										
Outcome variable	Obs	Score	Obs	Score	Difference	*p*	Obs	Score	Obs	Score	Difference	*p*	DiD	*p*
Job satisfaction	24	0.75	26	0.73	0.02	0.90	25	0.96	25	0.88	0.08	0.50	0.06	0.70
Pride	24	0.80	26	0.71	0.09	0.90	25	0.90	25	0.72	0.18	0.40	0.09	0.70
Intrinsic motivation	24	0.33	26	0.57	0.25	0.10	25	0.90	25	0.70	0.20	0.40	0.00	0.80
Timeliness and attendance	24	0.95	26	0.88	0.07	0.28	25	0.80	25	0.60	0.20	1.00	0.10	0.40
Organisational commitment	24	0.38	26	0.35	0.03	0.80	25	0.56	25	0.28	0.28	0.04	0.25	0.20
Overall motivation	24	0.63	26	0.73	0.11	0.40	25	0.80	25	0.72	0.08	0.50	0.01	0.90

DiD, difference-in-difference; Obs, observations.

In total, 30 of 33 intervention health workers reported their overall motivation as having improved since PBI implementation.

As for the allowances and the awards, it motivated us to work extra hard since we knew we were competing with others from other facilities. Getting something small at the end of the month also made me feel my work was appreciated. (Midwife, aged 60, intervention facility)

Among three workers reporting no improvement, the main reasons were that the PBIs fostered competition with their colleagues that stifled teamwork and that the PBIs were not equitable because only some benefited.

It made me to work as if I was in competition with other people, which I think is not the best and does not really keep you motivated. (Midwife, aged 33, intervention facility)

Since we know if we work hard we will be rewarded, we worked separately trying to prove that we are the best and this made it difficult to actually know how we would have worked if there were no awards. (Midwife, aged 60, intervention facility)

In comparison facilities, 32 of 33 workers reported their motivation as unchanged at the end of the study period.

Job satisfaction and general motivation are still a challenge in the provision of care. (Nurse, aged 26, comparison facility)

### Job satisfaction

Scores among health workers in intervention and comparison facilities at baseline were 0.75 and 0.73, respectively. End line scores increased to 0.96 in intervention facilities and 0.88 in comparison facilities, whereas the DiD was 0.06 (*p*=0.70) – neither of which were significant differences.

Improved job satisfaction was reported as a key benefit of PBIs by all intervention health workers.

My satisfaction with my work improved when I received a certificate of recognition, a blender and a cloth. (Midwife, aged 28, intervention facility)

### Pride

Scores among health workers in intervention and comparison facilities at baseline were 0.80 and 0.71, respectively. End line scores increased to 0.90 in intervention facilities while remaining at 0.72 in comparison facilities, and DiD was 0.09 (*p*=0.70) – neither of which were significant.

In total, 32 of 33 intervention health workers reported that PBIs helped them feel recognised as important stakeholders in the delivery of maternal care, which enhanced their pride. Meeting colleagues from different facilities and from the district level during award ceremonies and being recognised through an award were reported very positively.

At the award ceremonies, we are usually with our superiors from the district health directorate and as soon as we receive the awards in their presence and are congratulated afterwards, we become so proud as midwives working to achieve the MDGs. (Midwife, aged 54, intervention facility)

However, one health worker noted no improvement because she was already proud of her job as a midwife, helping women deliver healthy babies.

To be honest with you, I have not seen any change in my pride. I was already proud of conducting successful deliveries and so the PBIs did not do much to improve it. (Midwife, aged 58, intervention facility)

### Intrinsic motivation

Scores among health workers in intervention and comparison health facilities at baseline were 0.33 and 0.57, respectively, a non-significant difference (*p*=0.10). End line scores increased to 0.90 and 0.70 respectively, and DiD was 0.00 (*p*=0.8) – neither of which were significant.

All health workers reported that extrinsic rather than intrinsic motivation (e.g. rewards and competition with other facilities) inspired them to work harder.

It was very effective. It inspired us to work very hard, especially knowing that we will be judged and rewarded. (Midwife, aged 33, intervention facility)

The way I related to my patients also changed, I have become most friendly to them and I try to encourage them to come for ANC and delivery, since I know the more they come the more likely I am to get an award. (Midwife, aged 60, intervention facility)

### Timeliness and attendance

Scores among health workers in intervention and comparison facilities at baseline were 0.95 and 0.88, respectively (i.e. a non-significant difference of 0.07; *p*=0.28). End line scores decreased to 0.80 and 0.60 in intervention and comparison facilities, respectively, and DiD was 0.10 (*p*=0.40), neither of which were significant.

In total, 32 of 33 intervention health workers reported that PBIs motivated them to provide timely services. As performance was assessed through specific maternal health indicators, failure to report to work early resulted in an inability to meet PBI targets.

Knowing that I will be rewarded at the end of the day makes me punctual at work to attend to my clients in good time. (Midwife, aged 48, intervention facility)

I was always punctual to work because of the monthly allowance and awards such as tea a kettle, smock, blender, and television sets, which made me achieve our targets. (Male nurse aged 32, intervention facility)

However, one said the awards were too small to motivate her.

The awards given by the review committee were too small to motivate me to report early to work … (Midwife, aged 38, intervention facility)

Only one comparison health worker, whose facility was also participating in an intervention to improve emergency obstetric care, noted that timeliness and attendance had improved in her facility.

Motivation for work has changed slightly within the 2 years with regards to timeliness and attendance but more needs to be done about job satisfaction and organisational commitment as we lack protocols and incentives to work. (Midwife, aged 48, comparison facility)

### Allied and district-level staff

Reported perceptions supported those of health workers. Most suggested that PBIs motivated health workers to support service users, leading to fewer complaints at facilities.

The award is the external motivation; it created a platform for midwives to put up their best in my facility. (Facility Head, aged 55, intervention facility)

In terms of clients’ complaints, at first clients used to complain about the negative reception of health workers but since the PBI was introduced I did not hear any further complaints from the clients. (Facility Head, aged 60, intervention facility)

However, a few district-level staff mentioned negative effects, such as fostering competitiveness or potentially demotivating those who were not rewarded.

The award system served as a disincentive to workers who were not awarded. (Facility Head, aged 34, intervention facility)

Working while knowing in the back of your mind that you will be awarded is not all good. It made them work for the awards and not with passion. (District-level staff, aged 59, intervention facility)

District-level staff reported few changes in health worker motivation in comparison facilities.

… generally motivation of health-workers is still a problem. (District Director, aged 54, comparison facility)

### Organisational commitment

Scores among health workers in intervention and comparison facilities at baseline were 0.38 and 0.35, respectively. End line scores increased to 0.56 and remained at 0.28, respectively, a significant difference of 0.28 (*p*=0.04). DiD was not significant at 0.25 (*p*=0.20).

All intervention health workers reported enthusiastically that PBIs increased their commitment to their facility. Most indicated they were not considering leaving and would even recommend their profession to friends and relatives.

The award ceremonies create in me a desire to work more and more in this facility and so I don't consider moving out. (Nurse, aged 27, intervention facility)

If these incentives were given continuously to us in this facility, I would recommend to my relatives to also work in maternal health. (Midwife, aged 40, intervention facility)

In contrast, comparison health workers did not report changes.

There is not much improvement, especially in the area of general motivation and organizational commitment. Inadequate remuneration results in low motivation to work and this sometimes hinders the provision of effective and efficient healthcare in the facility. (Midwife, aged 32, comparison facility)

## Discussion

### Primary findings

This study of the effects of PBIs on constructs of maternal health worker motivation is, to the authors’ knowledge, a first in low- and middle-income countries. Though the quantitative sample size was small, potentially preventing detection of significant differences in motivation constructs, the overall findings are encouraging and suggest PBIs can improve primary health worker motivation in this low-income area of northern Ghana. Further research is needed to determine whether suggested improvements can be sustained and issues around competition and demotivation of non-awardees addressed. Research in Tanzania, indicating P4P improved health worker motivation and service outcomes, suggests that improved maternal health worker motivation could improve maternal health outcomes in northern Ghana (63–65).

Quantitative findings indicated that only one construct, organisational commitment, improved significantly among intervention versus comparison health workers. Other constructs that improved somewhat were job satisfaction, intrinsic motivation, and timeliness/attendance, though none were significant at the 0.05 level. Though these constructs were not significantly different from comparison values at end line, several had improved further from baseline than had comparison values, indicating that a larger sample might have detected significant differences. For example, commitment may have been significant while other constructs were not due to fewer response categories, enabling larger cell sizes and thus demonstration of significance.

Qualitative findings indicated that reported motivation had improved in the intervention facilities while remaining generally unchanged in the comparison facilities. Research in Rwanda indicated similarly that PBIs improved health worker motivation and control over service outputs (20, 66–68). Extrinsic motivation appeared key, as Patouillard et al. found that PBIs enhanced motivation among health workers with less intrinsic motivation (21). Improvement in timeliness in comparison facilities may have been influenced by the implementation of the Ghana Essential Health Project (GEHIP), which measured the timeliness of facility-based deliveries and emergency referrals (69).

### Implications

Ryan and Deci advocate incentives for any employee to focus on key obligations (70). This study attempted to fill a gap in the evidence by investigating the effects of PBIs on nurses and midwives in primary facilities in a low-income region of Ghana. The literature on PBIs indicates that health workers engage in self-monitoring when there is an incentive strategy and that they increase their productivity by spending more time doing incentivised work (71). The results are encouraging, despite the underpowered quantitative sample, indicating that PBIs may contribute to improving health worker performance within the Ghanaian health system (72, 73).

Unfortunately, the sample included in this study was small. Because this study does not contradict findings on PBIs in other countries, the authors recommend that the Ghana Health Service and Ministry of Health extend PBIs to other facilities and evaluate a larger sample over a longer period, to determine the long-term effectiveness and sustainability of PBIs at primary level. Further research into group/facility-wide incentives as opposed to individual incentives and support for intrinsic motivation (e.g. enjoyment and mastery) could help address health worker concerns about competing against each other. Additionally, economic analysis of PBI schemes should be conducted to determine whether they are worthwhile in resource-constrained settings such as northern Ghana. Potential national scale-up would depend on further research, including cost-effectiveness evaluation, which was not attempted in this study.

### Limitations

The potential study limitations are the cohort sample size (i.e. 25 per arm), potential contamination related to timeliness from external initiatives (e.g. GEHIP), and potential response bias in interviews. First, the sample included all primary-level maternal health workers in the two districts and was small because most maternity providers in Ghana work in hospitals. A larger sample, or possibly a longer implementation period (e.g. 4 years instead of 2 years), could have generated better statistical power. Because of the small sample size, rigorous quantitative analysis controlling for confounders (e.g. age, sex, and years worked in maternal health) could not be conducted and the representativeness is uncertain. Nevertheless, the DiD approach controlled for unobserved differences across the intervention and comparison facilities and the qualitative findings supported improved motivation. Second, potential confounding from the GEHIP study could have affected timeliness values, though differences were not noted in quantitative results. Third, some response bias was possible in interviews if participants exaggerated improved motivation constructs (e.g. playing a ‘good participant’ role). A larger blinded study would help dispel this concern. Despite potential limitations, this study is among the first to explore the effects of PBIs on health worker motivation in primary facilities in Ghana, providing a useful foundation for further research.

## Conclusions

The findings suggest that PBIs could improve motivation constructs among maternal health workers in Ghana, at least in the short term. If sustainable, this could contribute to improved maternal and neonatal health care in Ghana. Though the quantitative results are inconclusive, they serve as a first step. Overall, the results are sufficiently positive to contribute to advocacy for policy guidance on PBIs in Ghana, which is currently non-existent. Further research is needed with larger samples on the long-term effectiveness and sustainability of PBIs in improving motivation among frontline health workers in Ghana and similar countries.

## Paper context

Performance-based incentives (PBIs) can improve health worker motivation in low- and middle-income countries, but evidence is limited on the effects of PBIs on motivational constructs. This study contributes initial data on the effects of PBIs on motivational constructs among maternal health workers in northern Ghana. To determine the long-term effectiveness and sustainability of PBIs, the Ghana Health Service and Ministry of Health could extend PBIs to additional facilities, evaluating a larger sample over a longer period.
